# Long-term diet-induced, tissue-specific changes in (non)adipose triglyceride stores in obese patients with type 2 diabetes mellitus

**DOI:** 10.1186/1532-429X-13-S1-P352

**Published:** 2011-02-02

**Authors:** Jacqueline T Jonker, Marieke Snel, Sebastiaan Hammer, Arend E Meinders, Hanno Pijl, Ingrid M Jazet, Johannes A Romijn, Johannes WA Smit, Albert de Roos, Hildo J Lamb

**Affiliations:** 1 Leiden University Medical Center, Leiden, Netherlands

## Introduction

A very low calorie diet (VLCD) induces considerable weight loss in patients with type 2 diabetes mellitus, associated with improved insulin sensitivity and decreased triglyceride (TG) stores in (non)adipose tissues. Long-term effects of a VLCD on tissue-specific TG accumulation, including pericardial fat, have not been documented.

## Purpose

To assess the effects of a 16-week VLCD and of subsequent 14 months of follow-up on a regular diet on tissue-specific TG stores in obese type 2 diabetes patients.

## Methods

We included 14 obese patients with insulin treated type 2 diabetes (mean±SEM: age 53±2 years; BMI 35±1 kg/m^2^). (Non)adipose TG stores were measured using magnetic resonance (MR) imaging and MR proton spectroscopy before and after a 16-week VLCD (Modifast, 450 kcal/day) and after a 14-month follow-up without dietary interventions.

## Results

A 16-week VLCD significantly reduced bodyweight, hepatic TG content, visceral and subcutaneous abdominal fat and pericardial fat volumes to 78, 16, 40, 55 and 83%, respectively, of baseline values (all p<0.05). After an additional 14 months of follow-up on a regular diet, weight and hepatic TG content increased significantly to 90 and 73% of baseline values (P<0.05). After these 14 months the preferential loss in visceral fat compared to subcutaneous abdominal fat was lost. In contrast, pericardial fat volume remained reduced to the same extent.

## Conclusions

VLCD-induced weight loss and subsequent regain of weight during regular diet induces tissue-specific variations in (non)adipose TG stores in obese type 2 diabetes patients.

